# Multitargeting activity of miR-24 inhibits long-term melatonin anticancer effects

**DOI:** 10.18632/oncotarget.7978

**Published:** 2016-03-08

**Authors:** Federica Mori, Maria Ferraiuolo, Raffaela Santoro, Andrea Sacconi, Frauke Goeman, Matteo Pallocca, Claudio Pulito, Etleva Korita, Maurizio Fanciulli, Paola Muti, Giovanni Blandino, Sabrina Strano

**Affiliations:** ^1^ Molecular Chemoprevention Unit, Molecular Medicine Area, Regina Elena National Cancer Institute, 00144 Rome, Italy; ^2^ Translational Oncogenomics Unit, Molecular Medicine Area, Regina Elena National Cancer Institute, 00144 Rome, Italy; ^3^ Department of Research, Advanced Diagnostics and Technological Innovation, Translational Research Area, Regina Elena National Cancer Institute, 00144 Rome, Italy; ^4^ Department of Oncology, Juravinski Cancer Center-McMaster University, Hamilton, ON L8V 5C2, Ontario, Canada

**Keywords:** melatonin, miR-24, RNA-Seq, p53, PML

## Abstract

We have previously shown that melatonin exerts tumor suppressor activities by inducing the p38-p53 axis. This occurred within a few hours while no data are available on how melatonin pathway can be sustained on the long term. Here we show that miR-24, which has been demonstrated to target genes involved in the DNA repair process, targets p38, p53, PML and H2AX simultaneously. We show that long-term treatment with melatonin can decrease miR-24 levels post-transcriptionally, which pairs with a long-wave regulation of genes involved in cell proliferation, DNA damage, RNA metabolism and cell shape and transformation. Moreover, we show that melatonin can inhibit cell proliferation and migration, at least in part, by downregulating miR-24. Furthermore, we propose the involvement of hnRNP A1, which is downregulated by melatonin and involved in miRNA processing, in the regulation of miR-24 levels by melatonin. We conclude showing that miR-24 is upregulated in colon, breast and head and neck datasets and its levels negatively correlate with overall survival.

## INTRODUCTION

Melatonin exerts a plethora of activities, among which radical scavenging and activation of tumor suppressor pathways [1–12]. These activities explain in part the negative correlation observed between melatonin levels and the risk of developing cancer. In particular, it is worth to mention three epidemiological prospective studies conducted on healthy subjects, two of them on the ORDET cohort [13, 14] and the third one on the Nurses' Health cohort [15], which showed a negative correlation between the urinary 6-sulphatoxymelatonin (aMT6s) levels and breast cancer risk.

A few studies have shown that melatonin can activate phosphorylation cascades, mediated by MEK1/2, ERK1/2, JNK and p38 MAPK [10, 11, 16, 17], through binding to its G protein-coupled membrane receptors MT1 and MT2. This leads to the activation of tumor suppressor pathways, such as p53. In particular, melatonin treatment induces a p38-mediated phosphorylation of p53 in Ser15, which caused a transient cell cycle arrest and the accumulation of DNA repair proteins (e.g. H2AX and PML) and the induction of DNA repair mechanisms themselves [6, 10]. These phenomena occur only in the presence of intact MT1 and MT2 signalling [11].

These studies have been conducted on short intervals and showed fast phosphorylation kinetics, at least concerning p53. Although it is evident that melatonin activities are sustained over long periods of time (e.g. increase in p53 levels following 72 hours treatment with melatonin), no data are available on their long-term regulation. Being deregulated in many cancers and involved in the carcinogenic process [18–21], micro-RNAs (also known as miRNAs and miRs) are good candidates for long-term regulation of tumor suppressive pathways, such as p53. They are small non-coding RNAs [22], which regulate gene expression by inducing degradation or inhibiting translation of their numerous target genes [23]. They are transcribed as a long primary RNA (pri-miRNA or pri-miR), which usually contains multiple miRNAs. Before leaving the nucleus, pri-miRs are cleaved by Drosha and DGCR8 [24–27] into a smaller precursor RNA (pre-miRNA or pre-miR). pre-miR is then exported into the cytoplasm [28–30], where Dicer cleaves it into 22 nucleotides long double-stranded miRNAs [31, 32]. These are bound by the RNA-induced Silencing Complex (RISC), which catalyzes the separation of the two complementary strands, indicated as mature miR-5p and miR-3p (reviewed in [33]). Both strands are able to regulate gene expression and show different target genes [34]. They can also elicit sometimes completely different outcomes, as in the case of miR-10b and miR-10b*. Indeed, overexpression of miR-10b positively regulates cell migration and invasion in metastatic- and its overexpression initiates robust invasion and metastasis in non-metastatic breast tumors [35], while overexpression of miR-10b* associates with longer relapse- and metastasis-free survival [18, 36].

Being such an important process, miRNA processing is tightly regulated by several proteins. Apart from Dicer-binding proteins [25, 37–41], RNA-binding proteins concur to miRNA maturation. Heterogeneous Nuclear Ribo-Nucleo-Proteins (hnRNPs) have recently been shown to be involved in miRNA processing, the most known examples being probably the involvement of hnRNP A1 in the maturation of miR-18a [42, 43].

A variety of miRNAs regulate the DNA repair process by targeting DNA repair proteins [44], among which miR-24, which regulates histone H2AX expression, thereby causing genomic instability and reducing DNA repair [45]. miR-24 has been found in both pleural effusions and sera from patients with lung cancer, therefore it is now regarded to as a potential diagnostic tumor marker [46, 47]. In addition, miR-24 is upregulated in highly differentiated CD8+ T cells upon etoposide treatment and associated with a decreased DNA damage response [48] and in oral squamous cell carcinomas (OSCC) as compared to their matched controls [49], as well as in hepatocellular carcinomas (HCC), its expression being higher in higher grade HCC [50]. Moreover, miR-24 is overexpressed in glioblastomas and promotes cell growth and migration in glioma cells both *in vitro* and *in vivo* [51].

In the present manuscript, we show that melatonin downregulated miR-24, which targets four components of the melatonin activated pathway: p38, p53, PML and H2AX. By targeting these genes, miR-24 enhance cell proliferation and induce migration of cancer cells, while melatonin can revert these phenotypes in the presence of physiological levels of miR-24 but not when miR-24 is overexpressed by mimic transfection. We also show that miR-24 downregulation occur through intact MT1/MT2 receptor signalling, as it is abolished in the presence of the melatonin antagonist Luzindole. In addition, we propose the involvement of hnRNP A1 in the processing of miR-24, since miR-24 downregulation by melatonin seems to occur at the post-transcriptional level and melatonin downregulates hnRNP A1 expression through receptor signalling.

## RESULTS

### Melatonin downregulates miR-24 leading to upregulated expression of its targets

**Figure 1:**
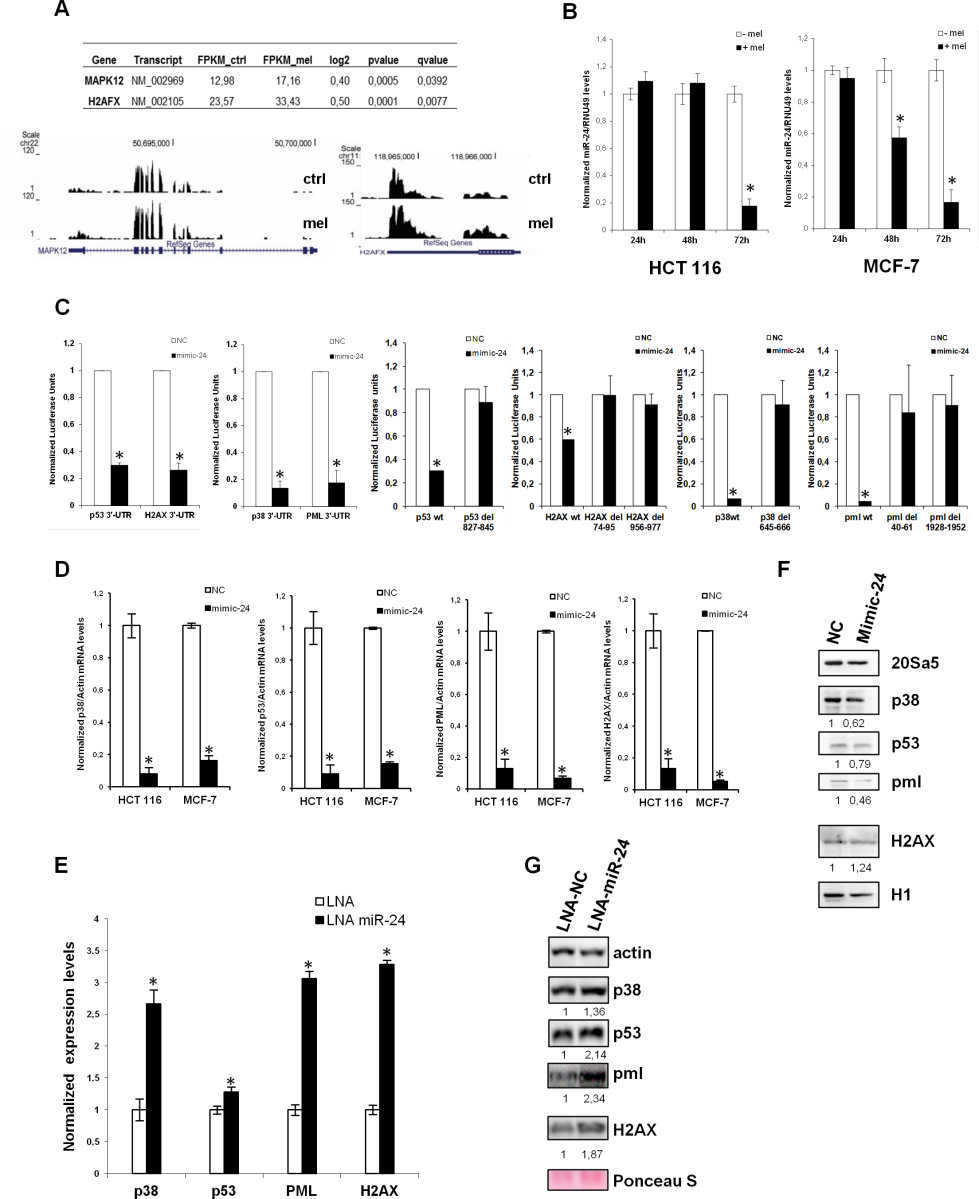
miR-24 targets the melatonin-p53 pathway (**A**) Graphs show the sequence coverage, as well as the signal intensity, of H2AFX (H2AX) and MAPK12 (p38-γ) in both vehicle and melatonin treated HCT 116 cell line. (**B**) HCT 116 and MCF-7 cells have been treated with melatonin for 24, 48 and 72 hours. The ratio between miR-24 and RNU49 levels, normalized to their respective untreated controls, are indicated in the graphs. (**C**) HCT 116 cells have been transfected with either negative control (NC) or miR-24 mimic and the indicated Dual Luciferase reporters. Values for NC are set to 1. *p < 0,01. (**D, E**) HCT 116 and MCF7 cells have been transfected with the indicated mimic (D) or LNA (E) and subjected to quantitative Real-Time PCR. Values have been normalized to NC or LNA. (**F, G**) Cells have been transfected with the indicated mimic (F) or LNA (G) and cell extracts have been subjected to immunoblot with the indicated antibodies. Numbers indicate signal quantification normalized to NC or LNA-NC.

**Figure 2:**
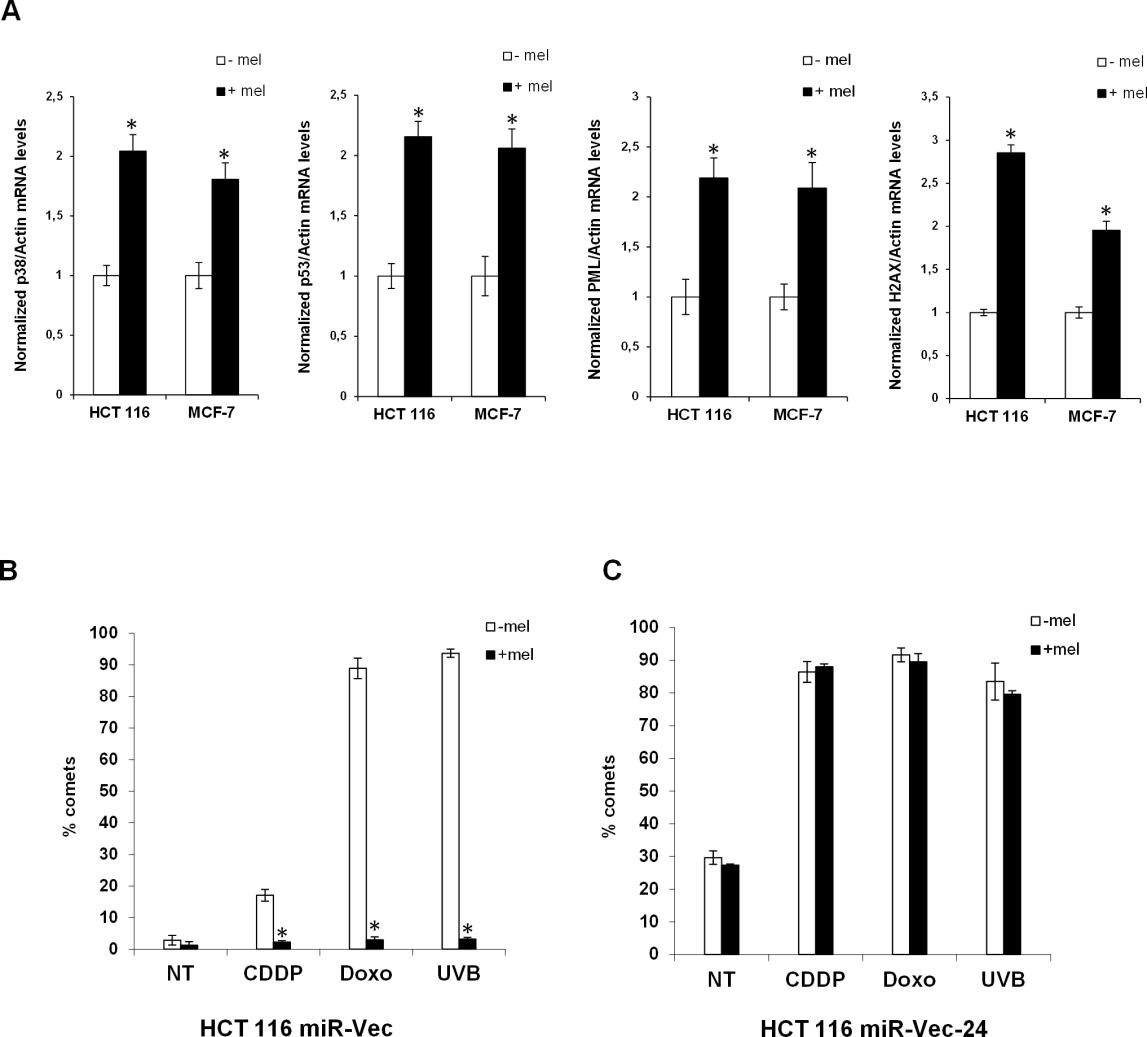
Melatonin induces the expression of the p53-pathway and alleviates genomic instability caused by miR-24 (**A**) HCT 116 and MCF-7 cell lines have been treated or not with melatonin for 72 hours and the expression levels of the indicated mRNAs were assessed by quantitative Real-Time PCR. Values have been normalized to untreated control. *p < 0,01. (**B**) HCT 116 cell lines stably expressing miR-Vec or (**C**) miR-Vec-24 have been pre-treated with either vehicle or melatonin, then DNA damage has been induced as indicated (CDDP: Cisplatin, Doxo: Doxorubicin, UVB: Ultra Violet B rays). Cells have been subjected to comet assay. Histograms show the percentage of comets.

We have previously shown that melatonin activates the p53 pathway with a fast kinetics through stimulation of its membrane receptors, MT1 and MT2 [10, 11]. However, it remained unclear how melatonin could sustain the p53 pathway activation on the long term, which would eventually lead to inhibition of cell proliferation and protection from DNA damage accumulation. To clarify this, we performed a search *in silico* to identify miRNAs predicted to regulate the four components of the melatonin pathway: p53, p38, PML and H2AX. We used the microRNA.org database and identified 60 miRNAs putatively targeting all four genes simultaneously. Among those, only five had validated targets in Tarbase: miR-24, miR-217, miR-296–5p, miR-324–3p and miR-876–3p (Table 1). miR-24 (alias miR-24–3p) raised our interest as it showed the highest number of validated targets, it had been previously reported to target H2AX and inhibit DNA repair [45]. It is also upregulated in a variety of cancers as compared to non-tumoral tissues (Supplementary Figure S1). We also interrogated the expression of predicted miR-24 putative target genes in a RNA-Seq experiment previously performed in our laboratory in vehicle and melatonin treated cells for 48 hours, to look at global changes in mRNA expression following melatonin treatment. We identified 14 upregulated genes following melatonin treatment which were also miR-24–3p putative targets (Table 2). Among the 14 genes we identified H2AX and p38-γ (Figure 1A). To assess whether miR-24 could be regulated by melatonin, we measured its expression levels after treatment with melatonin of both HCT 116 and MCF-7 cells. We found that miR-24 levels were strongly reduced following 72 hours of melatonin treatment in both cell lines and this appear already evident at 48 hours in MCF-7 cells (Figure 1B). This prompted us to validate our four genes of interest as targets of miR-24. We analyzed their expression upon miR-24 transfection by Dual Luciferase assays, Real-Time qPCR and Western Blot. Dual Luciferase assays confirmed that all four genes were direct targets of miR-24 (Figure 1C) and deletion of the seed sequence of miR-24 abolished their regulation by miR-24 (Figure 1C). Both Real-Time qPCR and Western Blot analyses showed that p53, p38 and PML expression was decreased upon transfection of miR-24 (Figure 1D, 1F). H2AX was downregulated by miR-24 at the mRNA but not protein level, probably due to the targeting of a specific 3′UTR variant of H2AX by miR-24. Conversely, inhibition of miR-24 expression by LNA transfection induced the expression of p38, p53, PML and H2AX both at the mRNA (Figure 1E) and the protein (Figure 1G) levels. miR-24 overexpressed and downregulated levels are monitored by qPCR (Supplementary Figure S2A–S2B). Strikingly, p38, p53, PML and H2AX transcripts are up-regulated in response to melatonin thus it is opposite to that of miR-24 upon the identical experimental conditions in both HCT 116 and MCF-7 cell lines (Figure 2A).

**Table 1:** miRNAs targeting the melatonin-p53 pathway

Micro-RNA	Validated targets
hsa-miR-24	217
hsa-miR-217	10
hsa-miR-296–5p	8
hsa-miR-324–3p	3
hsa-miR-876–3p	1

miRNAs targeting p38, p53, PML and H2AX have been predicted by microRNA.org. Only 5 out of 60 miRNAs targeting simultaneously the four genes had validated targets in Tarbase and have been listed in the Table according to the number of validated targets.

**Table 2:** miR-24–3p putative target genes regulated by melatonin

Gene	Transcript	FPKM_ctrl	FPKM_mel	log_2_	*p*-value	*q*-value
ATAD3B	NM_031921	2.47	3.62	0.55	0.0005	0.0371
CRLF1	NM_004750	1.95	3.16	0.69	0.0005	0.0371
H2AFX	NM_002105	23.57	33.43	0.50	0.0001	0.0077
MAPK12	NM_002969	12.98	17.16	0.40	0.0005	0.0392
MRPL27	NM_016504	56.10	72.29	0.37	0.0006	0.0449
NCLN	NM_020170	3.24	4.40	0.44	0.0006	0.0449
PRELID1	NM_013237	22.11	35.90	0.70	0.0001	0.0130
REEP6	NM_138393	5.00	7.21	0.53	0.0004	0.0312
RFC2	NM_181471	19.74	27.92	0.50	0.0004	0.0312
RPL10	NM_006013	56.79	76.97	0.44	0.0001	0.0077
RPS23	NM_001025	168.56	213.37	0.34	0.0002	0.0205
TAGLN	NM_003186	2.30	4.49	0.97	0.0001	0.0130
TMEM115	NM_007024	2.32	3.67	0.66	0.0001	0.0130
WDR18	NM_024100	6.60	9.63	0.55	0.0001	0.0077

miR-24 putative target genes upregulated following melatonin treatment in HCT 116 cells. FPKM, *p*- and *q*-values, as well as the log_2_ value, are shown.

### miR-24 causes genomic instability

We have previously reported that melatonin can reduce DNA fragmentation caused by chemotherapeutic administration through activation of the p38-p53 axis [10, 11]. Lal et al., have shown that miR-24 causes DNA damage through downregulation of histone H2AX [45]. We reasoned that, p53- and p38-dependent DNA repair induced by melatonin could be mediated by its ability to reduce miR-24 expression levels. To this purpose, we assessed DNA damage accumulation in HCT 116 cell lines stably overexpressing miR-24 (miR-Vec-24) or its negative control (miR-Vec). Our results confirmed that miR-24 could increase DNA damage induced by chemotherapy as assessed by comet assays (Figure 2B–2C). Interestingly, melatonin was able to reduce DNA fragmentation only when miR-24 was expressed at physiological levels (Figure 2B–2C). This suggests that melatonin's ability to protect cells from DNA damage is exerted, at least in part, through downregulation of miR-24 levels. miR-24 stably overexpressed levels are verified in qPCR analysis (Supplementary Figure S2C).

### Melatonin inhibits miR-24-mediated proliferation and migration

**Figure 3:**
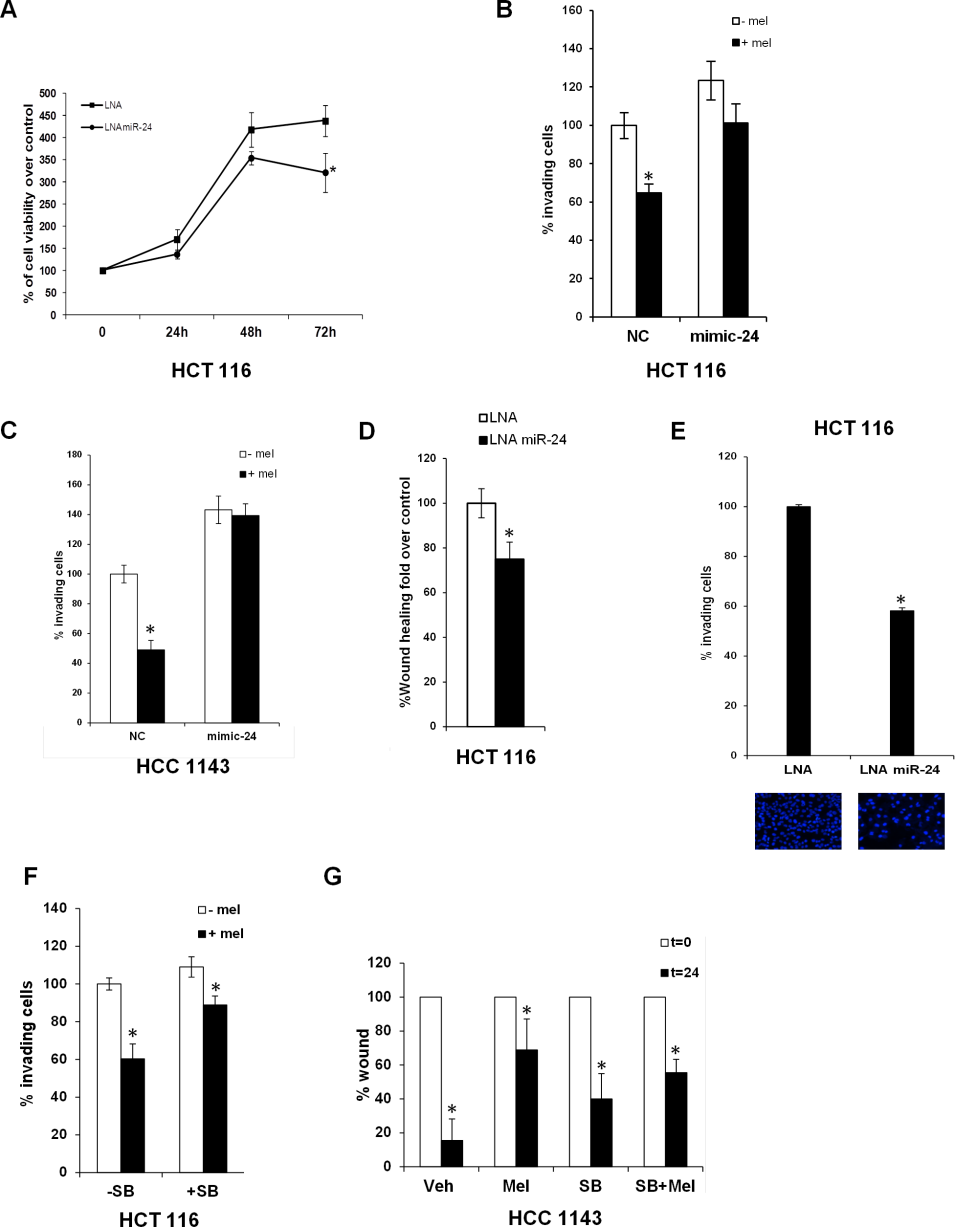
Melatonin impairs the capability of miR-24 to induce cell proliferation and migration (**A**) Cells were transfected with the indicated LNA and allowed to proliferate. Percentage of cells relative to control is indicated in the graph. *p < 0,05. (**B**) HCT 116 and (**C**) HCC1143 cells were transiently transfected with either NC or mimic-24 and subjected to transwell migration assays in the absence and in the presence of melatonin. Histograms show the percentage of invading cells. *p < 0,001. (**D**) HCT 116 cells were transfected with the indicated LNA and subjected to wound healing assay. Histograms show the percentage of wound opening. *p < 0,05. (**E**) HCT 116 were transfected with the indicated LNA and subjected to transwell migration assay. Histograms show the percentage of invading cells. *p < 0,01. (**F**) HCT 116 cells have been treated with 5 μM SB202190 for 2 hours and then subjected to transwell migration assay. *p < 0,05. (**G**) HCC1143 cells have been subjected to wound healing assay in the absence and in the presence of SB202190 and melatonin. Histograms show the percentage of wound opening. *p < 0,05.

Melatonin has been shown to inhibit cell proliferation through activation of p53 [6, 8, 10, 11]. We found that inhibition of miR-24 expression by LNA transfection reduced HCT 116 cells proliferation (Figure 3A). As miR-24 has been shown to regulate migration and invasion [52], we performed transwell migration assays in the presence or in the absence of melatonin in HCT 116 and HCC1143 cells transfected with either control or mimic-24. Melatonin inhibited cell migration at physiological levels of miR-24, which *per se* enhanced the percentage of migrating cells, while this ability was lost when miR-24 was overexpressed (Figure 3B–3C). Similar findings were found by LNA-mediated inhibition of miR-24 expression (Figure 3D–3E). miR-24 downregulated levels are verified in qPCR analysis (Supplementary Figure S2D). To understand whether melatonin and miR-24 regulated the invasive processes through the p38-p53 axis, we performed transwell migration assays in the presence and in the absence of the p38 inhibitor SB202190, which we previously showed to impair melatonin-induced p38-mediated phosphorylation of p53 [10]. Melatonin reduced partially the migration of HCT 116 cells in the presence of SB202190, but the extent of reduction was far lower (Figure 3F).

These results were corroborated by wound healing assays in HCC1143 cells: melatonin inhibited the wound healing process, while blockage of p38 impaired melatonin's ability to reduce wound closure (Figure 3G). Both sets of data suggest that, at least, part of melatonin inhibitory activity towards migration is exerted through the p38 signalling.

### Melatonin downregulates miR-24 through inhibition of hnRNP A1

**Figure 4:**
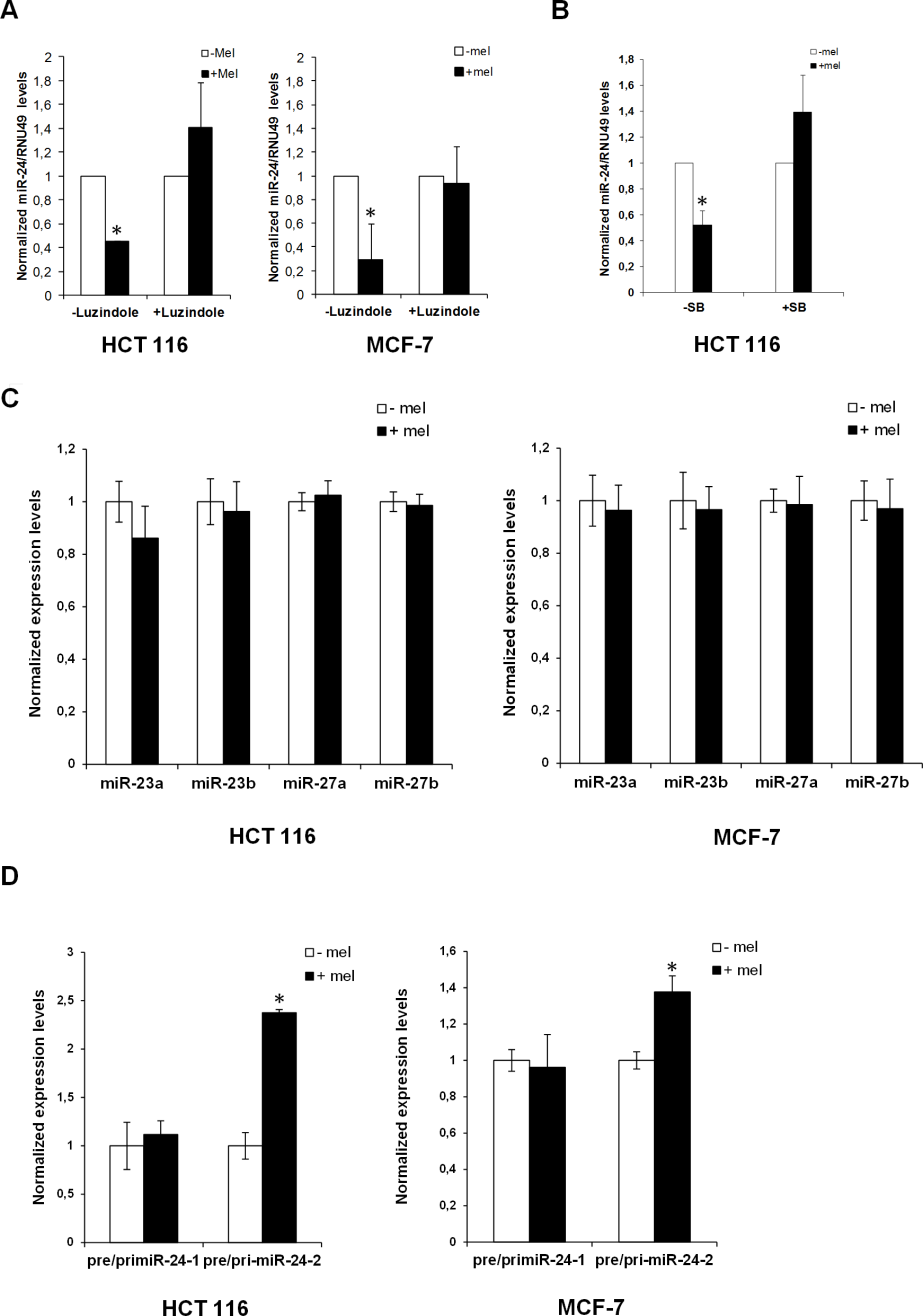
Regulation of miR-24 levels by melatonin (**A**) HCT 116 and MCF-7 cells have been either pretreated or not with Luzindole 15 minutes before melatonin treatment and miR-24 levels have been quantified 72 h later by quantitative Real-Time PCR. Histograms show miR-24/RNU49 levels relative to untreated control. *p < 0,01. (**B**) HCT 116 cells have been treated with melatonin for 72 hours in the absence and in the presence of 5 μM SB202190. Histograms show miR-24/RNU49 levels relative to untreated control. *p < 0,01. (**C**) HCT 116 and MCF-7 cells were treated with melatonin for 72 h. Levels of expression of the indicated miRNAs were assessed by quantitative Real-Time PCR. (**D**) HCT 116 and MCF-7 cells were treated with melatonin for 72 h. Histograms show the ratio between pre-miR-24–1 and pri-miR-24–1 and the ratio between pre-miR-24–2 and pri-miR-24–2 levels. *p < 0,01.

We reported that melatonin anticancer activities are exerted mainly through activation of MT1 and MT2 receptors [11]. To ascertain whether downregulation of miR-24 by melatonin could be exerted through MT1 and MT2, we investigated miR-24 expression levels following melatonin treatment in the absence and in the presence of the melatonin receptors inhibitor Luzindole. Quantitative Real-Time PCR revealed that blockage of melatonin receptors impaired melatonin's ability to reduce miR-24 levels (Figure 4A). Likewise, inhibition of p38 activity by treatment with SB202190 impaired miR-24 downregulation by melatonin (Figure 4B), suggesting that miR-24 expression was regulated through the p38-mediated melatonin receptor signalling.

miR-24 is transcribed from two different clusters: miR-23b∼27b∼24–1 and miR-23a∼27a∼24–2 [53]. To understand whether miR-24 was regulated at either the transcriptional or the post-transcriptional level by melatonin, we examined the expression levels of miR-23a, miR-23b, miR-27a and miR-27b. Interestingly their expression did not seem to vary upon melatonin treatment (Figure 4C), suggesting that the two clusters were not regulated transcriptionally. To corroborate these results, we amplified both pri-miR-24 and pre-miR-24 by Real-Time qPCR, both in the absence and in the presence of melatonin. Interestingly, while we could not appreciate a variation in the ratio between pre-miR-24–1 and pri-miR-24–1, the ratio between pre-miR-24–2 and pri-miR-24–2 increased upon melatonin treatment (Figure 4D), this may suggest that in the presence of melatonin pre-miR-24–2 would not be converted to mature miR-24.

**Figure 5:**
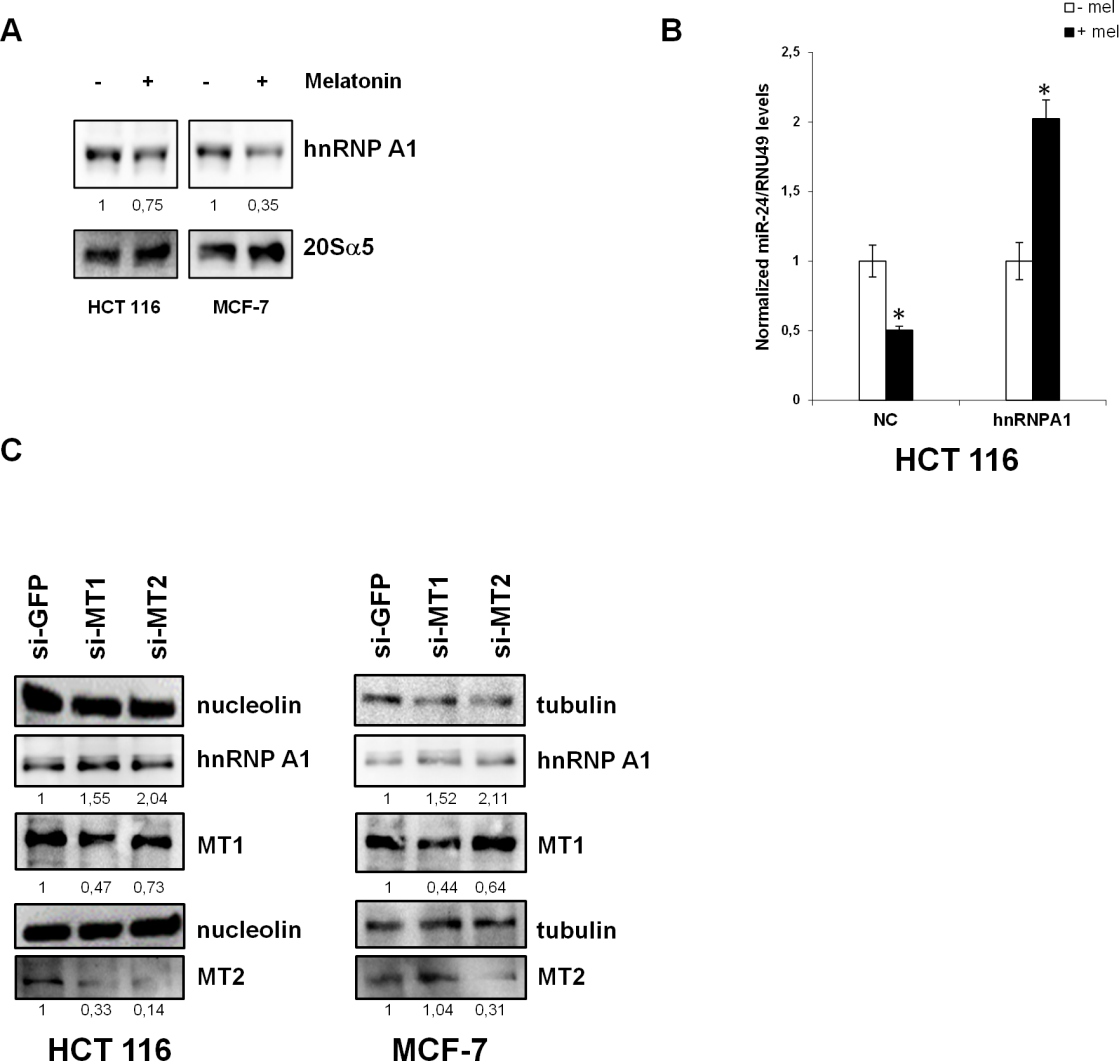
hnRNP A1 is involved in the regulation of miR-24 levels (**A**) HCT 116 and MCF-7 cells were treated with melatonin for 72 h and protein extracts were subjected to immunoblot with the indicated antibodies. Numbers indicate the densitometry ratio between hnRNP A1 and 20Sα5 signals. (**B**) HCT 116 cells were transfected with the indicated plasmids and treated with melatonin for 72 h, miR-24 levels were assessed by quantitative Real-Time PCR. *p < 0,01. (**C**) HCT 116 and MCF-7 were transfected with the indicated siRNAs. 72 h post transfection, cell lysates were subjected to immunoblot with the indicated antibodies. Numbers indicate the densitometry ratio between hnRNP A1 and tubulin or nucleolin signals, normalized to siGFP.

Given that melatonin treatment induced the expression of miR-24 target genes (Table 2), which are involved in cell proliferation (WDR18, PRELID1, CRLF1, TMEM115), cell shape and transformation (TAGLN, REEP6), RNA metabolism (RPS23, RPL10) and DNA damage response (RFC2, MAPK12, H2AFX), we reasoned that miR-24 downregulation could be a major event in melatonin anticancer activities. Therefore, we searched for a possible regulator of miR-24 maturation in a proteomic screening previously performed in our laboratory, to look at global changes in protein expression following melatonin treatment (data not shown). We found that hnRNP A1, a protein involved in both mRNA splicing and miRNA maturation [42, 43, 54–56], was downregulated upon melatonin treatment (data not shown). We confirmed this finding by Western Blot (Figure 5A). Of notice, hnRNP A1 mRNA levels were not affected by melatonin treatment (Supplementary Figure S3), suggesting a post-transcriptional regulation by melatonin.

To confirm hnRNP A1 involvement in miR-24 maturation, we overexpressed hnRNP A1 and treated the cells with melatonin. While melatonin led to a decrease in miR-24 at physiological levels of hnRNP A1, we could not observe a decrease in miR-24 when hnRNP A1 was overexpressed (Figure 5B), suggesting that melatonin-induced miR-24 downregulation was exerted through inhibition of hnRNP A1 expression. We then sought to analyze whether the decrease in hnRNP A1 levels occurred through activation of melatonin membrane receptors. To this purpose, we knocked down the expression of MT1 and MT2 by siRNA and investigated hnRNP A1 protein levels. Results showed that removal of either receptors led to an increase of hnRNP A1 protein levels (Figure 5C).

### miR-24 and hnRNP A1 are deregulated in human cancers

**Figure 6:**
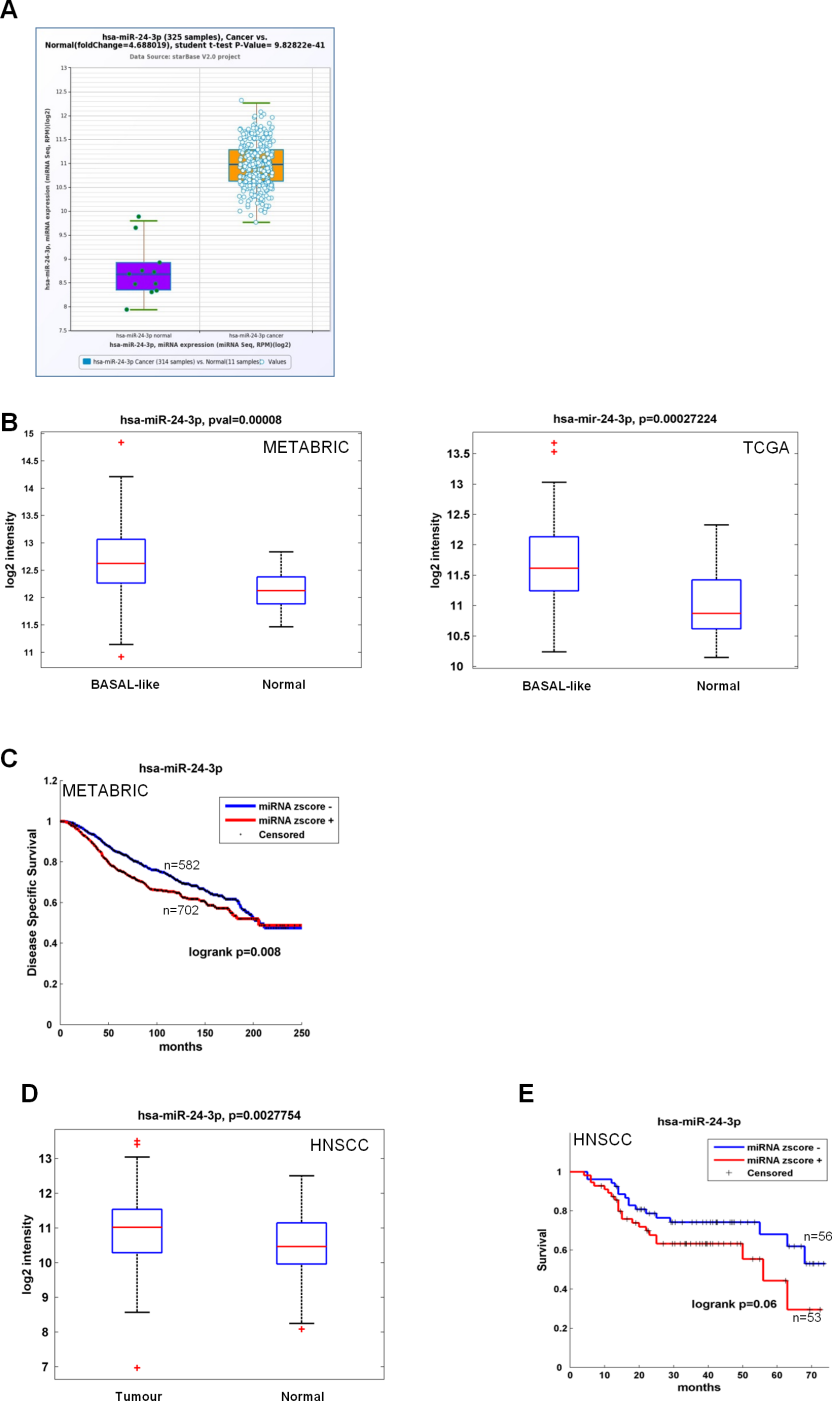
Clinical association of miR-24 with survival and recurrence in cancer patients (**A**) Box plot showing miR-24 median value in 314 cancer cases and 11 controls belonging to the Pan-Cancer Colon and rectal adenocarcinoma datasets. (**B-C**) Box plot showing miR-24 median value in 208 basal-like cancers and 116 controls (METABRIC) and 90 basal-like cancers and 83 controls (TCGA) (B) and association between expression levels of miR-24 and disease-free survival evaluated by Kaplan-Meier analysis in the METABRIC dataset, comprising 1284 cases and 116 controls (C). (**D–E**) Box plot showing miR-24 median value in 121 cancer cases and 66 controls (D) and association between expression levels of miR-24 and recurrence-free survival evaluated by Kaplan-Meier analysis (E) in our HNSCC casuistry [61].

We investigated miR-24 expression levels in both colon and breast cancer datasets. For colon cancer we used the Pan-Cancer colorectal adenocarcinoma dataset [57, 58], comprising 314 cases and 11 controls, while for breast cancer we interrogated both the METABRIC [59] and the TCGA [60] databases, comprising 1284 cases (208 basal-like) and 116 controls, and 694 cases (90 basal-like) and 83 controls, respectively. We found that miR-24 levels were far higher in tumor vs normal samples in both datasets for colorectal carcinoma (Figure 6A). We observed a statistically significant upregulation of miR-24 in the basal-like breast cancer subtype as compared to normal samples (Figure 6B) for breast cancer. Kaplan-Meier analyses in the METABRIC dataset showed that high miR-24 levels correlated with a lower disease specific survival rate (Figure 6C). To verify that miR-24 acted as an oncogene in diverse cancer types, we analyzed its expression from our head and neck squamous cell carcinomas dataset [61]. We found that, also in this dataset, miR-24 was upregulated in tumors as compared to non-tumoral matched samples (Figure 6D) and that high levels of miR-24 correlated with a lower survival (Figure 6E). These data, together with the proliferation and migration data shown in Figure 3, corroborate our and others finding that miR-24 acts as an oncogenic microRNA [52, 62].

A few reports have shown that hnRNP A1 is deregulated in human cancers [63, 64]. We verified the upregulation of hnRNP A1 in both colon and breast cancer, by analyzing its expression in four colon (Supplementary Figure S4A) and four breast (Supplementary Figure S4B) cancer datasets in Oncomine (www.oncomine.org) [59, 63–69]. We found it overexpressed in tumor vs normal samples. These data might suggest hnRNP A1 involvement in both colon and breast cancer.

## DISCUSSION

**Figure 7:**
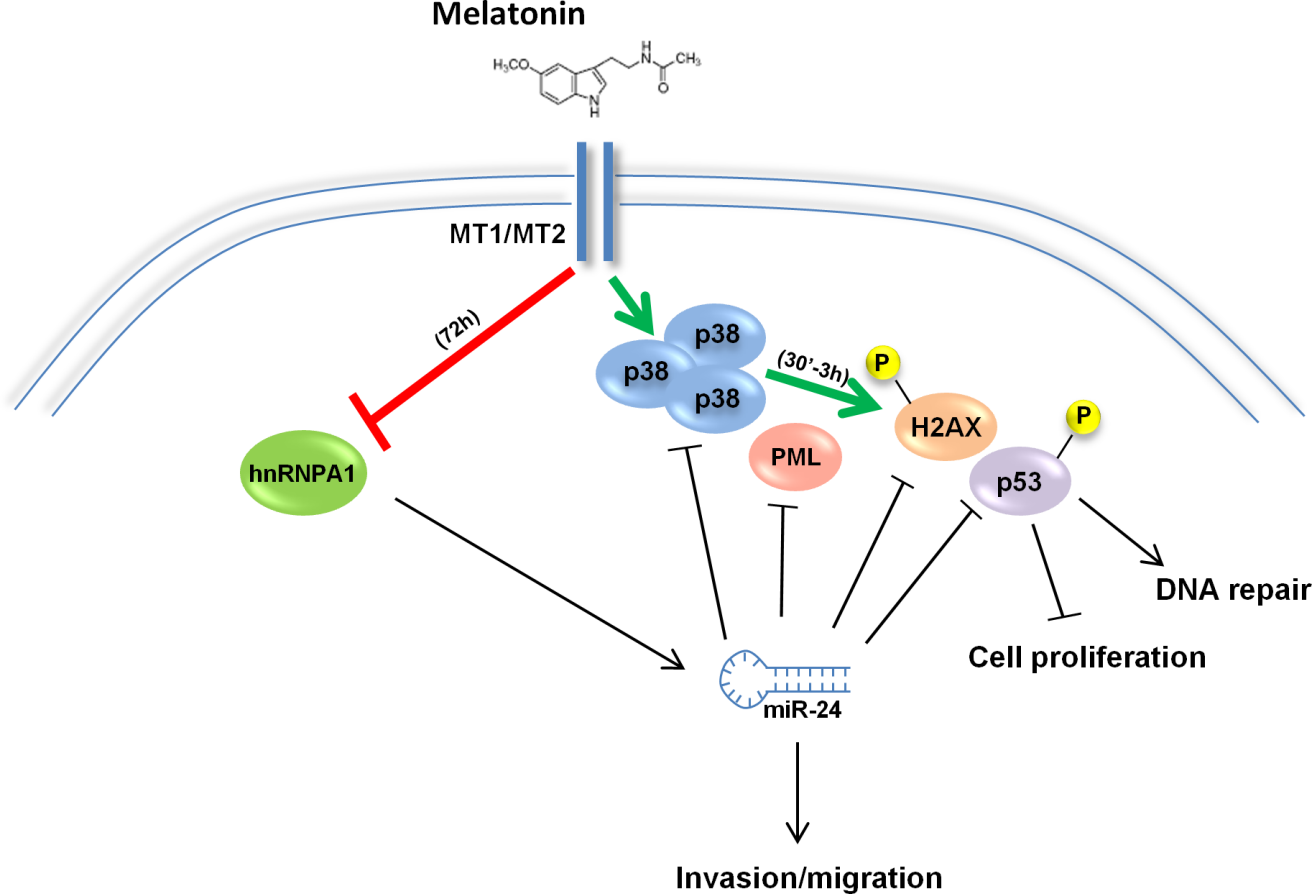
Model of melatonin activities By binding to its receptors (MT1/MT2), Melatonin induces p38 expression. With a fast kinetics (green arrow), Melatonin induces phosphorylation of p53 and H2AX thereby promoting DNA repair and inhibiting cell proliferation. Long-term activation of Melatonin receptors sustains p38 activation and leads to inhibition of hnRNP A1 (red arrow) thereby causing a decrease in miR-24, which results in p53, PML, H2AX and p38 activity reduction. Through downregulation of hnRNP A1 and miR-24, melatonin impairs the migrating capability of cancer cells.

Epidemiological studies have shown that melatonin levels negatively correlate with the risk of developing various kinds of cancers [13–15]. We have previously shown that melatonin can induce the p38-p53 axis through membrane receptor activation [10, 11]. However, while our previous data focused on a fast kinetics of induction of the p38-p53 axis occurring within 3 hours of treatment (Figure 7, green arrows), others have observed an upregulation of p53 at later time points [3, 6, 8] but there is no evidence of how melatonin can exert its long-term oncostatic effects. A possible mechanism could be miRNA regulation. However, at present there is only one report about miRNAs regulation by melatonin, in which the authors identified a set of miRNAs differentially modulated in untreated and treated MCF-7 cells [70] but neither the mechanisms by which melatonin can influence miRNA expression nor the mechanisms by which miRNAs can regulate melatonin's anticancer activities are known. Our work identifies the oncogenic miR-24 as a miRNA strongly repressed by melatonin 72 hours post treatment (Figure 7, red arrow), which has p38, p53, PML and H2AX among its target genes (Figure 1). In addition, we identified 14 putative targets of miR-24 after melatonin treatment (Table 2), among which H2AX and p38-γ. Of notice, H2AX had the highest mirSVR score (Supplementary Table S1), which could account for the fact that it was upregulated already 48 hours post treatment, when we observed a mild downregulation of miR-24 (Figure 1B). The decrease in miR-24 expression following melatonin treatment translated into activation of DNA repair and inhibition of cell proliferation and migration, due in part to the upregulation of the four key components of the melatonin receptor activated pathway (Figures 2 and 3). Thus, upon melatonin treatment, p38 induced a mechanism of self-sustainment through downregulation of miR-24 (Figure 4B). Our data on the melatonin-induced p38-dependent reduction of hnRNP A1 protein are supported by previous observations reporting that p38 can phosphorylate hnRNP A1 [71, 72], thereby changing its localization from the nucleus to stress granules in the cytoplasm, as well as reducing its protein levels [73]. Previous reports have also shown that hnRNP A1 can bind to miRNA precursors and regulate their cleavage acting as a miRNA chaperone [42, 43]. We suggest that hnRNP A1 mediates miR-24 processing in the absence of melatonin, as its ectopic expression abrogates miR-24 downregulation. We reported previously that melatonin membrane receptors MT1 and MT2 could be involved in the carcinogenic process, since their expression is lower in tumors as compared to normal samples [11]. Conversely, here we show that hnRNP A1 is upregulated in tumor vs normal samples in the same colon cancer casuistries where we observed repression of MT1 and MT2 and in an additional casuistry as well (Supplementary Figure S4A). This occurs also in four breast cancer casuistries deposited in Oncomine (Supplementary Figure S4B). Despite we show that miR-24 targets four crucial proteins involved in melatonin anticancer effects, this does not rule out the possibility that other target proteins as well as other miRs will play a major role in such activities.

miR-24 is regarded to as an oncogenic miRNA in various types of cancer because not only it targets DNA repair genes, such as H2AX [44, 45] but it has also been found upregulated in OSCC [49] (Figure 6D), hepatocellular carcinoma [50], glioblastoma [51] and breast cancer [74]. In addition, it is involved in the carcinogenic process by inducing transformation and migration [50, 51]. Our data provide evidence for both miR-24 oncogenic role in breast and colon cancer and melatonin inhibition of an oncogenic pathway. First, we provide evidence that miR-24 contributes to transformation of breast and colon cancer cell lines, as it conferred a higher proliferative and migratory potential to HCT 116 and HCC1143 cells and that its ectopic expression could overcome melatonin repression of these oncogenic features (Figure 3). Then, we show that miR-24 is upregulated in colon cancer, as well as in breast cancer. In particular, its expression levels are higher in colorectal adenocarcinoma as compared to normal samples in the Pan-Cancer casuistry, and in the basal-like subtype of breast cancer in the METABRIC and TCGA datasets (Figure 6A–6B). Moreover, we evaluated the association between expression levels of miR-24 and recurrence-free survival by Kaplan-Meier analysis and found a strong negative correlation for breast cancer (Figure 6C). As for breast cancer, we found that miR-24 was overexpressed in head and neck tumors and higher levels correlated with a worse prognosis (e.g. worse disease-free survival) (Figure 6D–6E). This suggests that miR-24 deregulation could be a carcinogenic event common to various types of cancer. Recently, miR-24 has been identified as a circulating miRNA in lung cancer patients. It has been found, together with miR-30d, as a cell-free miRNA in both malignant and benign pleural effusions, with higher levels in the malignant ones [47], as well as in pre-operation sera from lung cancer patients [46]. In the light of these observations, miR-24 could be used as a potential cancer biomarker.

These data strengthen the role of melatonin as a chemopreventive agent. In fact, we can speculate that not only melatonin could induce DNA repair (Figure 2B–2C) [75], thereby restraining the side effects of radio- and chemo-therapy on normal cells, but it could *per se* reduce migration and invasion, through downregulation of miR-24, thus ameliorating the prognosis of invasive cancers. Obviously, this speculation needs to be validated by assessing the impact of melatonin treatment on the DNA repair of untransformed cells. The use of melatonin as an adjuvant for both chemo- and radio-therapy could therefore be envisioned in patients with metastatic cancers and could be used as a therapeutic approach in tumors harboring p53 wild-type. In aggregate, it emerges that melatonin treatment instigates the activation of a tumor suppressor network, which finely tunes transcriptional events and non-coding mRNAs activity. The outputs of such network might depend from both the type and the stage of a given tumor.

## MATERIALS AND METHODS

### Cell culture and transfection

HCT 116 and MCF-7 cells (ATCC, Manassas, US) were cultured in DMEM supplemented with 10% FCS, 2 mM L-Glutamine, 100 Units/ml Penicillin and 100 μg/ml Streptomycin (Gibco, Life Technologies, Carlsbad, CA, US). HCC1143 cells were cultured in DMEM-F12 supplemented with 10% FCS, 100 Units/ml Penicillin and 100 μg/ml Streptomycin. Cell lines were grown at 37°C, 5% CO2. Transfections were performed with Lipofectamine 2000 and Lipofectamine RNAiMax (Life Technologies, Carlsbad, CA, US) according to the manufacturer's recommendations, using either negative control (NC) (cat. 4464061, Life Technologies, Carlsbad, CA, US) or mimic-24 (MC10737, Life Technologies, Carlsbad, CA, US) or LNA-control (LNA) or LNA-miR-24–3p (LNA miR-24) (Exiqon A/S, Vedbaek, Denmark). MT1 and MT2 receptors were silenced in HCT 116 cell line using siRNAs: siGFP 5′-AAGUUCAGCGUGUCCGGGGAG(dTdT)-3′; siMTNR1A 5′-GGAAUACAGGAGAAUUAUA(dTdT)-3′; siMTNR1B 5′-CUAGCUACUUACUGGCUUA(dTdT)-3′ (Eurofins MWG, Ebersberg, Germany). HCT 116 stable cell lines were generated by stable transfection of HCT 116 cells with either miR-Vec or miR-Vec-24 and selected for 2 weeks with 2 μg/ml Blasticidin (15205 Sigma-Aldrich, Saint Louis, Missouri, US).

### Reagents

Melatonin (1 μM, M5250), Luzindole (1 nM, L2407) and SB 202190 (5 μM, S7067) were purchased from Sigma-Aldrich (Saint Louis, Missouri, US).

### Mutant constructs

Plasmid mutant constructs have been generated by deleting miR-24 seed sequences in the parental Dual Luciferase Reporter constructs by Quick Change mutagenesis (Agilent Technologies, California, US) following manufacturer's instructions. The following primers have been used:

p38 del 645–666 FW: 5′-GTTAAAAGACTGCAGCGGGCAAGTCGAGAGGG-3′; p38 del 645–666 Rev: 5′-CCCTCTCGACTTGCCCGCTGCAGTCTTTTAAC-3′; H2AX del 74–95 FW 5′-ACCACCGCCCTCATGGAAAGCTTCAGACTGC-3′; H2AX del 74–95 Rev 5′-GCAGTCTGAAGCTTTCCATGAGGGCGGTGGT-3′; H2AX del 956–977 FW 5′-GATACCAGCAGAAGTCGGTTAA TCCGTTGGCTTCT-3′; H2AX del 956–977 Rev 5′-AG AAGCCAACGGATTAACCGACTTGTGCTGGTATC-3′; PML del 40–61 FW 5′-GGGATGGGGTCCAGGCCCCA CCCA-3′; PML del 40–61 Rev 5′-TGGGTGGGGCCT GGACCCCATCCC-3′; PML del 1928–1958 FW 5′-GCA GTCTGAAGCTTTCCATGAGGGCGGTGGT-3′; PML del 1928–1958 Rev 5′-GGATTCACATGCTTAAGAGGGCTCTCAGCTCTGC-3′; p53 del 827–845 FW 5′-GAGTGGAGTGGCGTTTTGCCTCCCCGGC-3′; p53 del 827–845 Rev 5′-GCCGGGGAGGCAAAACGCCA CTCCACTC-3′.

### Growth curves

HCT 116 cell line, transfected with either LNAcontrol (LNA) or LNA-miR-24–3p (LNA miR-24) (Exiqon A/S, Vedbaek, Denmark) were allowed to proliferate and viability was measured at 24 h, 48 h and 72 h by ATPlite assay (Perkin Elmer, Whaltman, MA, US), according to manufacturer's instructions.

### Dual luciferase assays

5 × 10^3^ cells were reverse transfected with either negative control or miR-24 mimic into 12-well dishes; 48 hours later, they were transfected with the indicated reporter constructs. After additional 24 hours, cells were lysed and subjected to Dual Luciferase assays on a GloMax 96 Microplate Luminometer (Promega, Madison, WI 53711 US), using the Dual Luciferase Reporter assay system (E1910, Promega, Madison, WI 53711 US). For the psiCheck2-based H2AX (kind gift of Dr. Ashish Lal) and p53 (kind gift of Prof. Moshe Oren) reporter constructs (Promega, Madison, WI 53711 US), having the 3′UTR of interest downstream the Renilla Luciferase, experiments have been conducted in triplicate and graphs show the ratio between Renilla and Firefly luciferase. As for the pEZX-MT01-based p38 and PML reporter constructs (Genecopoeia, Rockville, US), having the 3′UTR of interest downstream the Firefly Luciferase, experiments have been conducted in six replicate and values for Firefly Luciferase have been reported in the graphs. Student *t*-test has been used to calculate *p*-values.

### Western blots

Protein extracts were prepared as previously described [11]. All protein extracts were quantified by Bradford assay and equal amounts were loaded onto SDS–PAGE, transferred to polyvinylidene fluoride membranes (PVDF, Immobilon-P, Merck-Millipore, Life Science, Darmstadt, Germany) and subjected to immunoblot with the indicated antibodies. Antibodies to β-actin (A2288, AC-74, Sigma-Aldrich, Saint Louis, Missouri, US), 20Sα5 (ab11437 AbCam, Cambridge, UK), Tubulin (Ab18251 AbCam, Cambridge, UK), Nucleolin (ab13541, 4E2, Abcam, Cambridge, UK), MTNR1A (MT1) (ab87639, AbCam, Cambridge, UK), MTNR1B (MT2) (ab128469, AbCam, Cambridge, UK) p38 (9212, Cell Signaling, Danvers, MA, US), PML (sc5621, H-238, Santa Cruz Biotechnology, CA, US), p53 (sc126, DO1, Santa Cruz Biotechnology, CA, US), hnRNP A1 (8443, D21H11, Cell Signaling, Danvers, MA, US), H1 (sc34464, Santa Cruz Biotechnology, CA, US), H2AX (#2595 Cell Signaling, Danvers, MA, US) were diluted in 5% bovine serum albumin in Tris-buffered-saline/0.1% Tween-20. Secondary anti-mouse, anti-goat and anti-rabbit antibodies were purchased from Bio-Rad (Bio-Rad, Hercules, CA, US). Images were acquired using a VersaDoc MP instrument (Bio-Rad, Hercules, CA, US).

### Comet assays

Following transfection with the indicated constructs, cells were treated with melatonin for 2 hours and then subjected to DNA damaging treatments with CDDP (850 nM, TEVA, Italia, Italy), Doxorubicin (10 nM, Ebewe, Pharma, Austria) and 5-Fluorouracile (1,5 μM, Hospira, Illinois, US), or Ultra Violet B (UVB) irradiated at a dose of 0,05 J/cm2 using a Bio-Sun irradiation apparatus (Vilbert Lourmat, Marne-la-Vallée, France) and allowed to repair DNA for 4 hours. After treatment, cells were detached with trypsin and subjected to comet assay as described previously [10, 11]. DNA was stained with propidium iodide (Sigma-Aldrich, Saint Louis, Missouri, US) and pictures were taken using 60x magnification on an Axiovert 200 M microscope and Axiovision acquisition program (Zeiss, Oberkochen, Germania). At least 300 cells were scored for each slide.

### Transwell migration assays

Transfected cells were detached and counted. 5 × 10^4^ cells, in a volume of 100 μl DMEM containing 0,1% FBS, were seeded in the upper chamber while the bottom chamber of the transwell was filled with 600 μl of DMEM with 10% FBS. Either melatonin or ethanol was added to the upper chamber. Cells were allowed to migrate for 20 hours. Then cells remaining in the upper chamber were scrubbed away with a cotton pad and cells remaining on the bottom layer of the upper chamber were subjected to DAPI staining as follows: the upper chamber was washed twice with PBS and then cells were fixed with 4% formaldehyde for 20 minutes, permeabilized with 0,2% Triton for 25 minutes and, after being washed with PBS, they were stained with 5 μM DAPI for 5 minutes. Membranes were cut and mounted on microscopy slides. All the cells on each membrane were counted. Each sample was assayed in triplicate. Graphs show percentage of cells relative to control. Student *t*-test has been used to calculate *p*-values.

### RNA extraction, reverse transcription and quantitative real-time PCR

RNA was extracted with Trizol (Life Technologies, Carlsbad, California, US) following manufacturer's instruction and quantified using Nanodrop (Thermo Scientific, Waltham, Massachusetts, US). For mRNA, 500 ng RNA were reverse-transcribed with M-MLV reverse transcriptase (Life Technologies, Carlsbad, California, US) following manufacturer's instruction and 1/10 of the reverse transcription was subjected to Real-Time PCR using FAST SYBR Green mastermix (4385612, Life Technologies, Carlsbad, California, US). The following primers were used:

p53 FW 5′-GTCTGGGCTTCTTGCATTCT-3′; p53 Rev 5′-AATCAACCCACAGCTGCAC-3′; p38 alpha FW 5′-GTGCCCGAGCGTTACCAGAAC-3′; p38 alpha Rev 5′-CTGTAAGCTTCTGACATTTC-3′; PML FW 5′-CGCC CTGGATAACGTCTTT-3′; PML Rev 5′-ACTGTGGCTG CTGTCAAGG; H2AX 3′UTR FW 5′-AGCAAACTCA ACTCGGCAAT-3′; H2AX 3′UTR Rev 5′-ACTCCCCA ATGCCTAAGGTT-3′; beta actin FW 5′-GGCATGGGTC AGAAGGATT-3′; beta actin Rev 5′-CACACGCAGCT CATTGTAGAAG-3′; hnRNP A1 FW 5′-ATACTTTGCA AAACCACGAAACC-3′; hnRNP A1 RV 5′-CCTGCTA AGCTTTGTTTCCTAATTAAA-3′; pri-miR-24–1 FW 5′-GCGGTGAACTCTCTCTTGTA-3′; pri-miR-24–1 RV 5′-TTACAGACACGAAGGCTTTT-3′; pre-miR-24–1 FW 5′-GGTGCCTACTGAGCTGAT-3′; pre-miR-24–1 RV 5′-CTGTTCCTGCTGAACTGAG -3′; pri-miR-24–2 FW 5′-GTTCACAGTGGCTAAGTTCC-3′; pri-miR-24–2 RV 5′-ATCTCTGCTCCAAGCATCA-3′; pre-miR-24–2 FW 5′-GTGCCTACTGAGCTGAAA-3′; pre-miR-24–2 RV 5′-CTGTTCCTGCTGAACTGAG-3′.

miRNA were reverse transcribed from 10 ng RNA using the TaqMan^®^ microRNA Reverse transcription kit (4366596, Life Technologies, Carlsbad, California, US). 1 μl of the reaction was used for Real-Time PCR. Amplification was carried out using specific miRNA probes and the TaqMan^®^ Universal PCR Master Mix, no AMP erase UNG (4364343, Life Technologies, Carlsbad, California, US) in a 7500 Fast Real Time instrument (Life Technologies, Carlsbad, California, US). Student *t*-test has been used to calculate *p*-values.

### RNA-Seq

Total RNA from melatonin or vehicle treated HCT 116 cells was isolated using miRNeasy including the optional DNase treatment (Qiagen, Valencia, CA, US). RNA quality was verified using an Agilent Bioanalyzer (Agilent Technologies, Santa Clara, CA, US; RNA 6000 Nano kit). All RNAs used for subsequent library preparation had an RNA integrity number greater than 9.0. RNA libraries for sequencing were generated according to the Epicenter ScriptSeq v2 RNA-Seq library preparation kit with an initial ribosomal depletion step using Ribo-Zero Magnetic Gold. Starting material was 5 μg of total RNA. Based on qPCR quantification, libraries were normalized to 1 nM and denatured by using 0.1 N NaOH. Cluster amplification of denatured templates was carried out according to manufacturer protocol (Illumina, Inc., San Diego, CA, US). Sequencing was performed on a Genome Analyzer IIx (Illumina, Inc., San Diego, CA, US) in paired-end mode, sequencing 2 × 75 bp. For each sample generated by the Illumina platform, a pre-process step for quality control has been performed to assess sequence data quality and to discard low quality reads.

For the analysis we exploited the RNA-Seq analysis workflow RAP [76], that comprises of read mapping, transcript assembly and abundancy estimation followed by transcript-based differential expression via the Tuxedo suite [77]. Paired-end reads were mapped to the human genome assembly hg19 with TopHat and further analyzed by the Cufflinks-Cuffdiff pipeline to identify differentially expressed transcripts. We run the pipeline without novel transcript discovery, correcting for multiply mapping reads with the “-u” option of Cuffdiff and a mask file for rRNAs and tRNAs. The RNA-Seq was conducted with three biological replicates.

Putative miR-24–3p targets were identified using the predictive target search of miRWalk [78] with the programs DIANAmT, miRanda, miRDB, miRWalk, RNA22 and Targetscan, taking only those genes in consideration that were predicted to be miR-24–3p targets by at least 3 different programs. Differentially expressed genes and transcripts that are putative miR-24–3p targets are reported in Table 2, along with *p*-value and *q*-value as reported by Cuffdiff.

### Statistical analyses

No statistical method was used to predetermine sample size. Experiments were not randomized. For biochemical experiments we performed the experiments at least three independent times. Experiments for which we showed representative images were performed successfully at least 3 independent times. No samples were excluded from the analysis. The investigators were not blinded to allocation during experiments and outcome assessment. All *p*-values were determined using two-tailed *t*-tests and statistical significance was set at *p =* 0.05. The variance was similar between groups that we compared. For head and neck patients' samples, the ethical committee of the “Regina Elena” National Cancer Institute (protocol CE/379/08) approved the study and informed consent was obtained.

## SUPPLEMENTARY MATERIALS FIGURES AND TABLE


