# Supplementary material for: Multitargeting activity of miR-24 inhibits long-term melatonin anticancer effects
Source: Oncotarget. 2016 Mar 8;7(15):20532–48. doi: 10.18632/oncotarget.7978 (PMC4991473; doi:10.18632/oncotarget.7978)
Supplement: Supplementary file 1 [file oncotarget-07-20532-s001.pdf]

Multitargeting activity of miR-24 inhibits long-term melatonin anticancer effects

Supplementary Materials

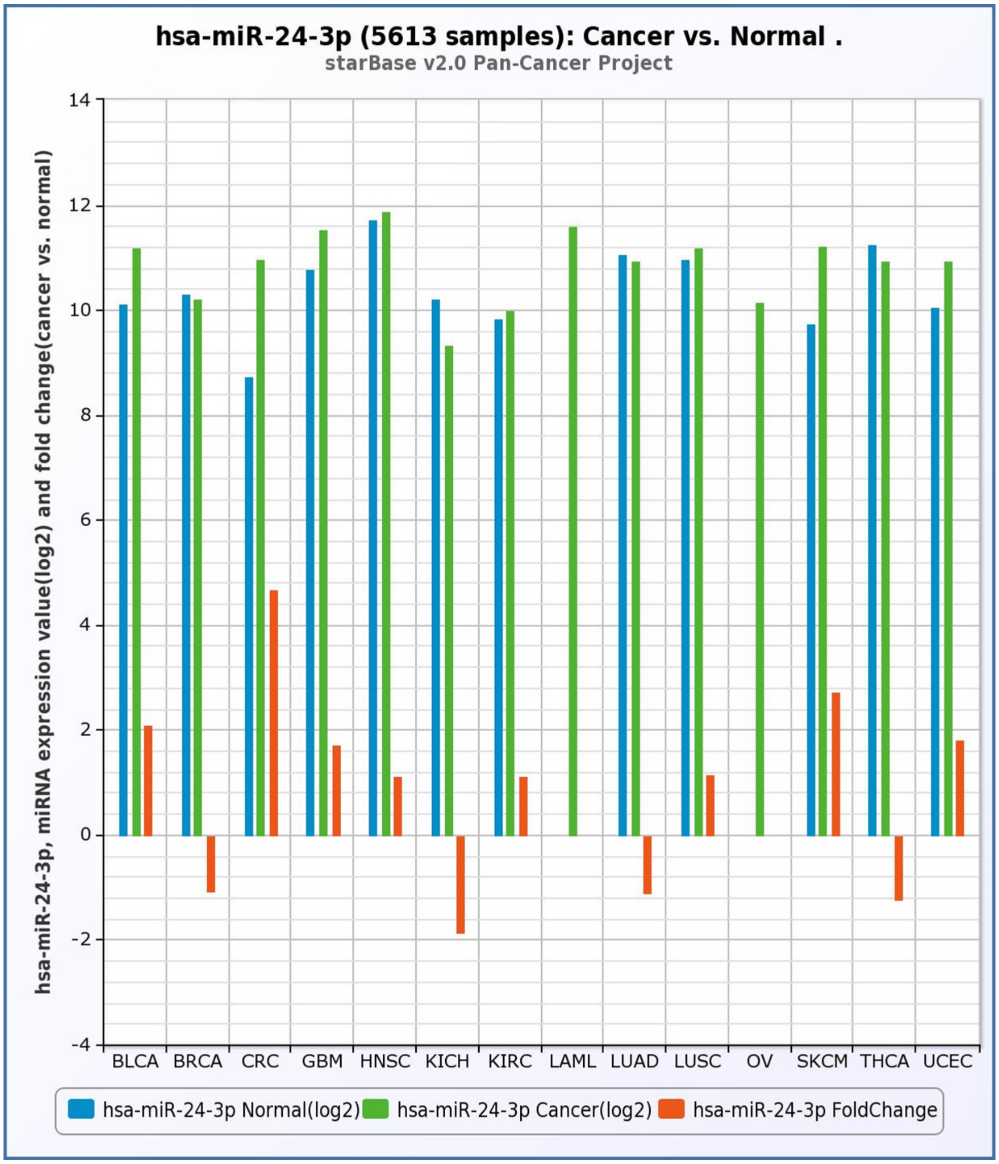

Supplementary Figure S1: Histograms show miR-24 levels in normal (blue bar) and cancer (green bar) tissues and the fold change for miR-24 expression in cancer vs normal tissue (red bar) in pan-cancer casuistries. BLCA: Urothelial bladder cancer; BRCA: Breast cancer; CRC: Colon and rectal adenocarcinoma; GBM: Glioblastoma multiform; HNSC: Head and neck squamous cell carcinoma; KICH: Chromophobe renal cell carcinoma; KIRC: Clear cell kidney carcinoma; LAML: Acute myeloid leukemia; LUAD: Lung adenocarcinoma; LUSC: Lung squamous cell carcinoma; OV: Ovarian serous cystadenocarcinoma; SKCM: Cutaneous melanoma; THCA Papillary thyroid carcinoma; UCEC: Uterine corpus endothelial carcinoma.

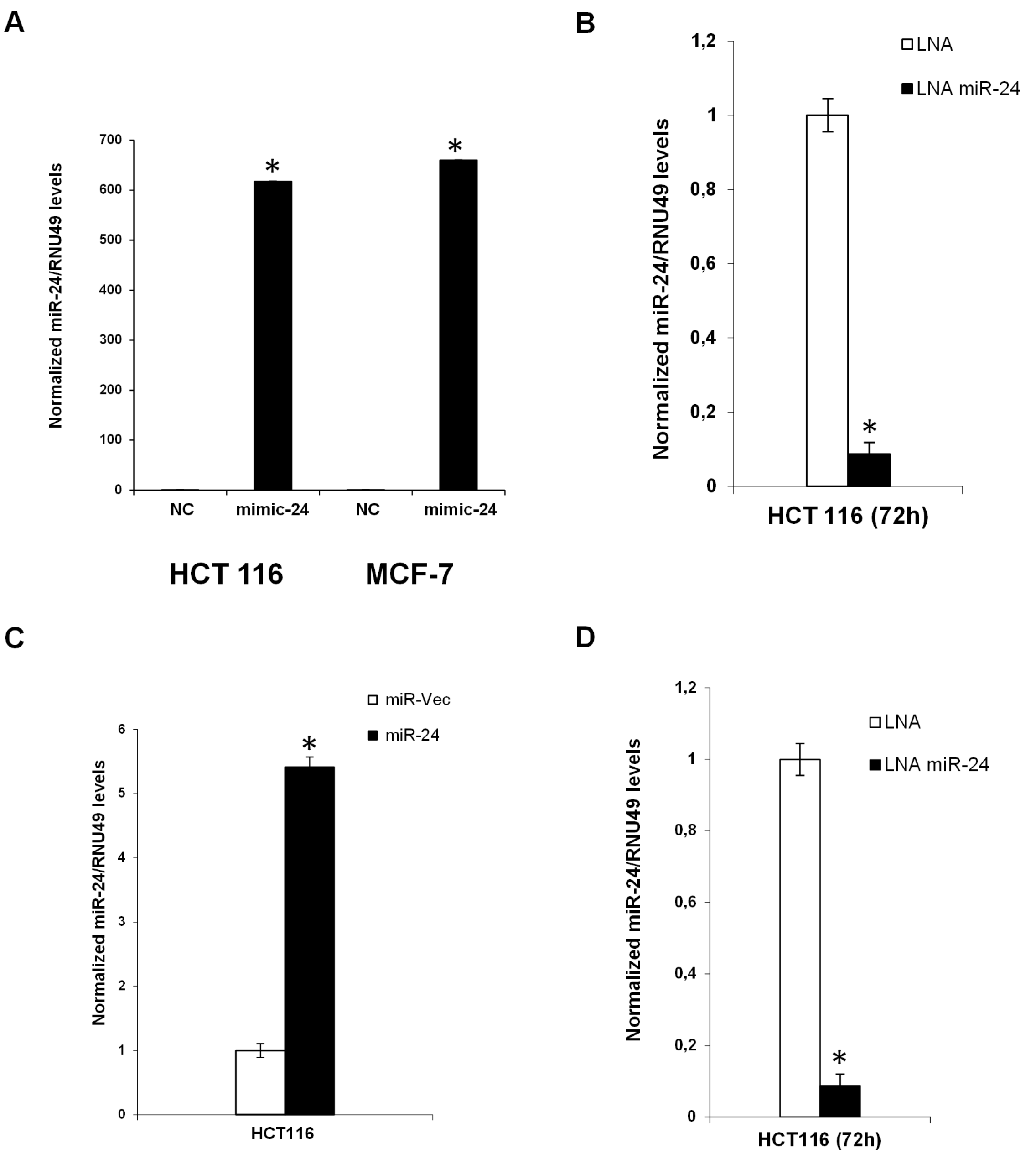

Supplementary Figure S2: (**A**) HCT 116 and MCF-7 cells were transfected with either negative control (NC) or mimic-24 and miR-24 levels were assessed by quantitative Real-Time PCR. **p* < 0,001. (**B, D**) HCT 116 cells were transfected with either LNA or LNA miR-24 and subjected to quantitative Real-Time PCR. Histograms show the ratio between target gene and RNU-49. Values have been normalized to LNA. **p* < 0,001. (**C**) HCT 116 cell lines were stably transfected with miR-Vec and miR-Vec-24, miR-24 levels are normalized on RNU-49 and value for miR-Vec is set to 1. **p* < 0,001.

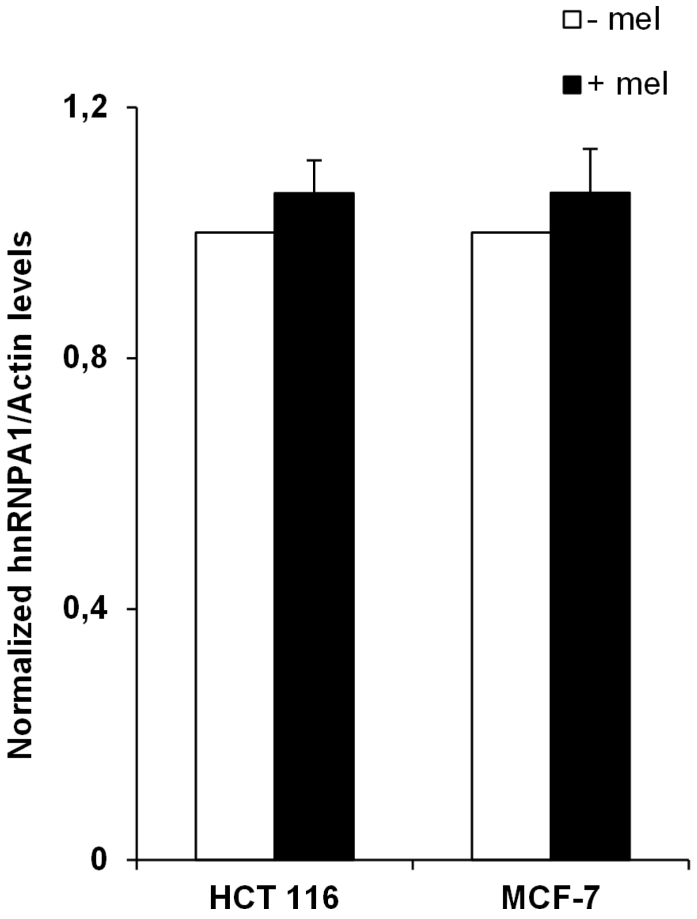

Supplementary Figure S3: HCT 116 and MCF-7 cells were treated or not with melatonin for 72 h and total RNA was subjected to quantitative real-time PCR. Histograms show the ratio between hnRNP A1 and beta-actin mRNA levels, normalized to untreated controls.

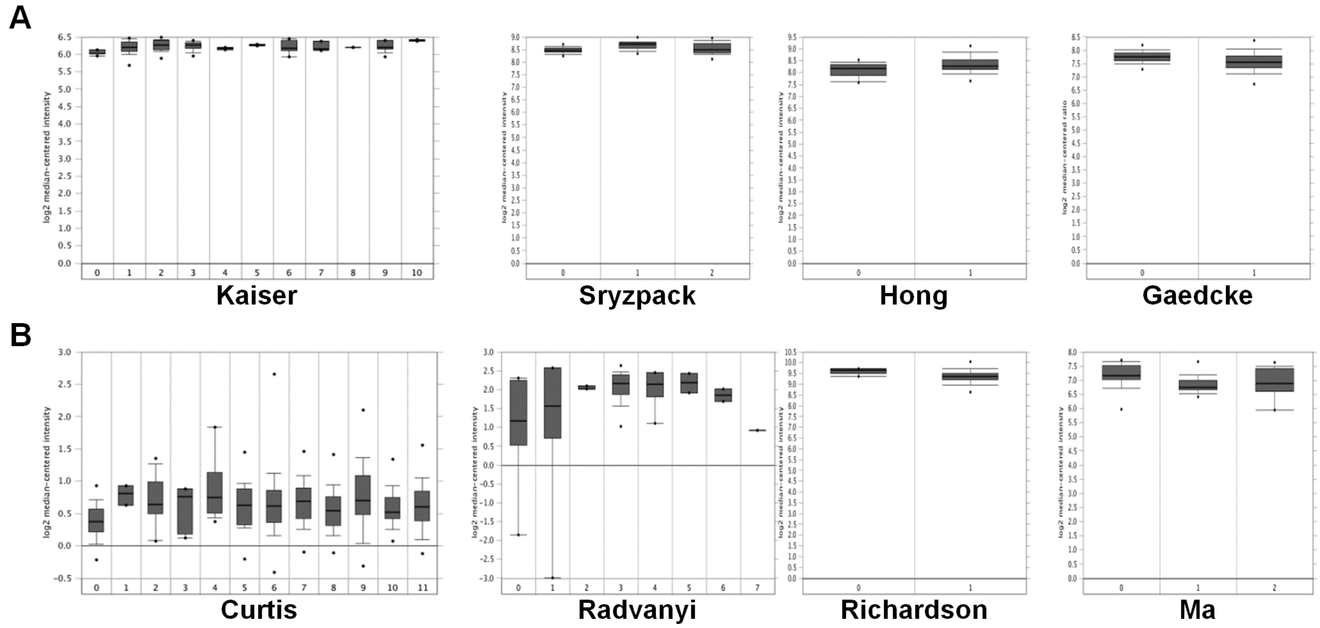

Supplementary Figure S4: hnRNP A1 is overexpressed in human cancers. (**A–B**) hnRNP A1 mRNA expression levels in colon (A) and breast (B) casuistries. (A) Kaiser casuistry. Kaiser Colon. Genome Biol 2007/07/05, 105 samples, hnRNP A1 Information: mRNA, 19,574 measured genes. Reporter Information: Human Genome U133 Plus 2.0 Array. Skrzypczak casuistry. Skrzypczak Colorectal. PLoS One 2010/10/01, 105 samples, hnRNP A1 Information: mRNA, 19,574 measured genes. Reporter Information: Human Genome U133 Plus 2.0 Array. Hong casuistry. Hong Colorectal. Clin Exp Metastasis 2010/02/01, 82 samples, hnRNP A1 Information: mRNA, 19,574 measured genes. Reporter Information: Human Genome U133 Plus 2.0 Array. Gaedcke casuistry. Gaedcke Colorectal. Genes Chromosomes Cancer 2010/11/01, 130 samples, hnRNP A1 Information: mRNA, 19,189 measured genes. Reporter Information: Agilent Human Genome 44K. (B) Curtis (METABRIC) casuistry. Curtis Breast. Nature 2012/04/18, 2,136 samples, hnRNP A1 Information: mRNA, 19,273 measured genes. Reporter Information: Illumina HumanHT-12 V3.0 R2 Array. Radvanyi casuistry. Radvanyi Breast. Proc Natl Acad Sci USA 2005/08/02, 63 samples, hnRNP A1 Information: mRNA, 16,775 measured genes. Reporter Information: Platform not pre-defined in Oncomine. Richardson casuistry.Richardson Breast 2. Cancer Cell 2006/02/01, 47 samples, hnRNP A1 Information: mRNA, 19,574 measured genes. Reporter Information: Human Genome U133 Plus 2.0 Array. Ma casuistry. Ma Breast 4. Breast Cancer Res 2009/02/02, 66 samples, hnRNP A1 Information: mRNA, 19,139 measured genes. Reporter Information: Affymetrix Human X3P Array.

Supplementary Table S1: miR-24 binding sites and mirSVR scores

3′UTR	miR-24 binding site	mirSVR score
**H2AX**	74	–0.1215
**H2AX**	956	–0.1664
**p53**	826	–0.1091
**p53**	1028	–0.0117
**p38**	645	–0.0921
**PML**	40	–0.0239
**PML**	1928	–0.0022

For each gene, the binding site and the mirSVR score are shown. H2AX, p53 and PML have 2 binding sites for miR-24 in their 3′UTR.
